# Tumefactive fibroinflammatory lesion presenting with head and neck fibrosclerosing lesions and orbital pseudotumors: a case report

**DOI:** 10.1186/1752-1947-7-260

**Published:** 2013-11-14

**Authors:** Soichi Kusaka, Sho Nishimura, Fumi Kawakami, Chiho Ohbayashi, Yasuyuki Shibuya, Kentaro Iwata

**Affiliations:** 1 Division of Infectious Diseases, Kobe University Hospital, Kusunokicho 7-5-2, Chuoku, Kobe, Hyogo 650-0017, Japan; 2 Department of Diagnostic Pathology, Kobe University Graduate School of Medicine, Kobe, Japan; 3 Department of Diagnostic Pathology, Nara Medical University, Nara, Japan; 4 Department of Oral and Maxillofacial Surgery, Kobe University Graduate School of Medicine, Kobe, Japan

**Keywords:** Inflammatory pseudotumor, Tumefactive fibroinflammatory lesion

## Abstract

**Introduction:**

Tumefactive fibroinflammatory lesion is an idiopathic fibrosclerosing disorder occurring in the head and neck region. It is one of a broad spectrum of entities named inflammatory pseudotumors and, as the name suggests, it mimics a lot of diseases such as malignancies or infections. Combined with its rarity, tumefactive fibroinflammatory lesion can be a tremendous diagnostic challenge. This case report describes a case of tumefactive fibroinflammatory lesion, which was initially thought to be peri-orbital and mandibular osteomyelitis caused by *Aspergillus*. A lengthy work up ensued and was required to reach the final diagnosis.

**Case presentation:**

A 64-year-old Asian man with a history of diabetes mellitus and chronic kidney disease who was on hemodialysis presented with worsening exophthalmos and relapsing trismus. He was diagnosed as “mandibular osteomyelitis” about 20 years ago. Since then he had suffered chronic relapsing exophthalmos and jaw pain with numerous medical treatments. In 2011 he was diagnosed as peri-orbital and intramandibular aspergillosis because a serum *Aspergillus* galactomannan assay was positive. He was treated with multiple antifungal medications to no avail. A biopsy of his orbital lesions was not revealing. After repeated biopsies, we finally concluded that the patient was suffering from tumefactive fibroinflammatory lesion. Corticosteroid therapy was initiated with prompt response.

**Conclusions:**

Tumefactive fibroinflammatory lesion is a rare inflammatory benign tumor, which mimics many inflammatory and neoplastic disorders. Conventional work up including biopsy may not lead to the diagnosis without understanding this entity. Awareness of this disorder will aid early diagnosis and treatment.

## Introduction

Tumefactive fibroinflammatory lesion (TFIL) is a rare idiopathic fibrosclerosing disorder occurring in the head and neck region, with a benign histological appearance [1]. It is one of a broad spectrum of entities called inflammatory pseudotumors [2]. Rice *et al*. first described the abnormality in 1975 and named this as sclerosing cervicitis [3]. Histologically identical lesions were subsequently reported involving parotid gland, nasal and paranasal sinuses, maxillary antrum, infratemporal fossa, lung, and extremities [4-7]. This lesion clinically simulates a malignant neoplasm without metastasis, but is histologically benign. Its histopathologic appearance is nearly identical to that of fibrosclerotic lesions of the mediastinum and retroperitoneum, as well as of Riedel’s thyroiditis [4]. The etiology of TFIL is unknown. In this case report we describe a case of TFIL in a Japanese man whose condition remained undiagnosed for about 20 years.

## Case presentation

A 64-year-old Asian man was referred to the Division of Infectious Diseases with a past medical history of trismus, exophthalmos, and positive serum *Aspergillus* galactomannan assay. His past medical history included diabetes mellitus type 2 for 25 years and chronic kidney disease on hemodialysis for 10 years.

About 20 years prior to the current presentation, he was diagnosed with “mandibular osteomyelitis” and treated with unknown antibiotics. About 10 years prior to the presentation, he was reported to have suffered candidemia. At that time, a radioisotope bone scan showed an uptake in his mandibular and he was treated with unknown antibiotics. His symptoms of “mandibular osteomyelitis” had waxed and waned since then. About 8 years prior to the presentation, another recurrence of “mandibular osteomyelitis” occurred and multiple antibiotics were provided including clarithromycin, levofloxacin, minocycline and fluconazole.

About 2 years prior to the current presentation, swelling of his left eyelid developed and it spread to his cheek. A computed tomography (CT) scan of the area revealed a lesion consistent with inflammation around his mandibular joint and surrounding soft tissue. Left exophthalmos with pain developed 2 months later. Magnetic resonance imaging of the area found an intra-orbital mass-like lesion and a lesion consistent with inflammation around his left masseter muscle. His visual acuity was slightly declining (from 20/30 to 20/100). Most symptoms improved to some extent without specific treatment but his visual acuity remained unchanged.

Bilateral mandibular pain developed 2 months prior to the current presentation (September 2011). Levofloxacin was provided without improvement. On the following month, an ophthalmological examination revealed swelling of his left eyelid, exophthalmos, and impaired visual acuity (from 20/100 to 20/220). He was referred and admitted to the Division of Infectious Diseases service because of an elevated *Aspergillus* galactomannan level (0.5 optical density (OD) index; reference normal level <0.5 OD index) in December 2011.

On admission, he was alert and his vital signs were: temperature 36.7, pulse rate 79/minute regular, blood pressure 143/63mmHg, and respiratory rate 12/minute. He had trismus and was able to open his mouth only by a few centimeters. There was no muscle spasm or fasciculation. His left eyelid was swollen with exophthalmos. The light reflex of his left eye was slow and weak. Supraduction of his left eye was impaired. There were no tender to touch areas on his face. His left vision was 20/220. The rest of his physical examination was unremarkable. A blood test revealed elevated *Aspergillus* galactomannan level (0.5 OD index), C-reactive protein level (8.6mg/dL) and erythrocyte sedimentation rate (86mm/hour) but was otherwise unremarkable, including a β-D-glucan assay which was <3pg/mL (reference range <20.0pg/mL). Imaging studies demonstrated no evidence of pancreatitis, mediastinal or retroperitoneal fibrosis, Riedel’s thyroiditis or Mikulicz’s disease.

**Figure 1:**
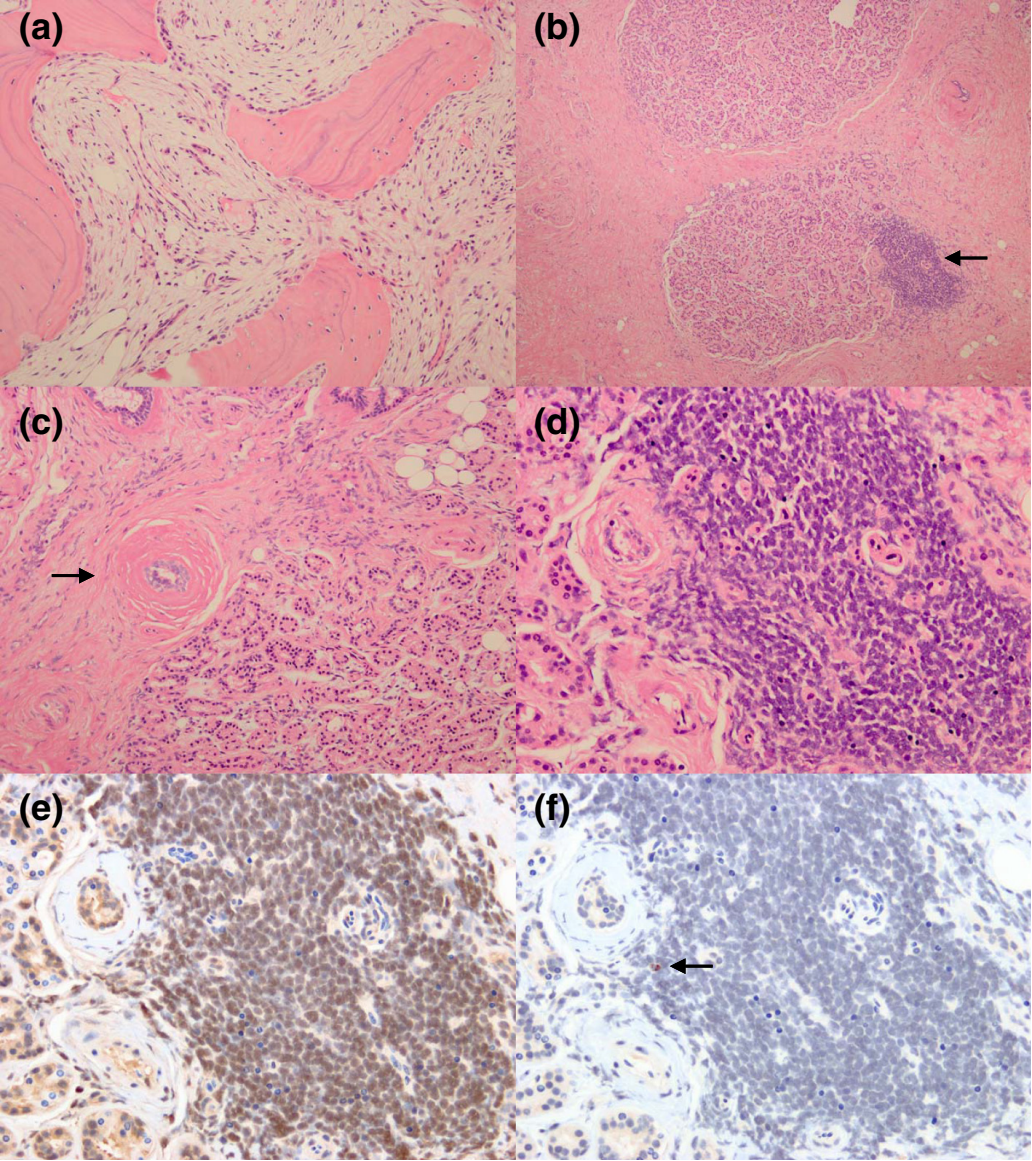
**Histology of the lachrymal gland and mandibular bone.** Fibrous inflammation characteristic of tumefactive fibroinflammatory lesion. **(a)** Hematoxylin and eosin staining of mandibular bone. Trabeculae were lined by osteoblasts showing active bone remodeling, and intertrabecular spaces were filled with fine fibrous tissue. There was mild lymphocytic infiltration, but no evidence of active osteomyelitis. **(b) (c) (d)** Hematoxylin and eosin staining of lachrymal gland. **(b)** Lachrymal glands and ducts were surrounded by sclerotic fibrous stroma (arrow). **(c)** The lobules are slightly atrophic. Periductal sclerotic fibrosis (arrow) was seen. **(d)** Lymphocytic aggregate was seen at the periphery of the lobule. **(e) (f)** Immunohistochemical staining for IgG **(e)** and IgG4 **(f)** of lachrymal gland. IgG4-positive cells (arrow) were very few.

Intravenous liposomal amphotericin B (300mg/day or 5mg/kg/day) and oral voriconazole (400mg/day) combination was initiated for presumptive chronic osteomyelitis caused by *Aspergillus*. In addition, dexamethasone (6mg/day) was provided to try to reduce inflammation to protect his vision. However, his visual acuity continued to decline and exophthalmos worsened gradually. On the 4th day after the admission, a surgical debridement was performed to decompress the optic canal and the orbit. Upon surgery, his extra-ocular muscles and eyeball were almost intact. However, there was thickening of the periostea of the bones surrounding his orbit up to 7mm. The lesion appeared inflamed and had a greenish yellow color on visual inspection. The staining and culture of debrided specimens were negative for bacteria, mycobacteria, and fungi. At histology, mildly atrophic lachrymal glands and ducts were surrounded by sclerotic fibrous stroma. Fibrosis was arranged in a concentric pattern around the ducts (periductal fibrosis). Although there were several foci of lymphocytic aggregation at the periphery of the lobules of his lachrymal glands, plasma cells or eosinophils were not dominant and only a few immunoglobulin (Ig) G4-positive cells were identified immunohistochemically. Typical obliterative phlebitis was not seen (Figure 1). The patient’s visual acuity, trismus, and exophthalmos partially improved after the operation. He was discharged home 15 days after the admission. Voriconazole was continued without corticosteroids.

Two months after the admission, exophthalmos and trismus on the right (opposite) side developed, accompanied by profound fatigue and appetite loss. He was readmitted to the hospital. His *Aspergillus* galactomannan level was 0.3 OD index. Liposomal amphotericin B was restarted and voriconazole was continued. However, he had exophthalmos, his visual acuity declined and his headache worsened. Dexamethasone was restarted (6mg/day) on the day of admission to rescue his visual acuity. Another operation for decompression was performed 10 days after the second admission, and this time, specimens were not sent for pathological examination due to a communication error. His symptoms improved since the operation. Dexamethasone was tapered off after 4 weeks. He was again discharged home on oral voriconazole.

A month after the second discharge (April 2012), however, another episode of trismus occurred. He was again hospitalized on May 1, 2012. He was not able to open his mouth at all because of trismus. There was severe tenderness on his bilateral orbits, maxilla and mandible. There were also lymphadenopathies on the left side of his neck with diameters up to 15mm.

**Figure 2:**
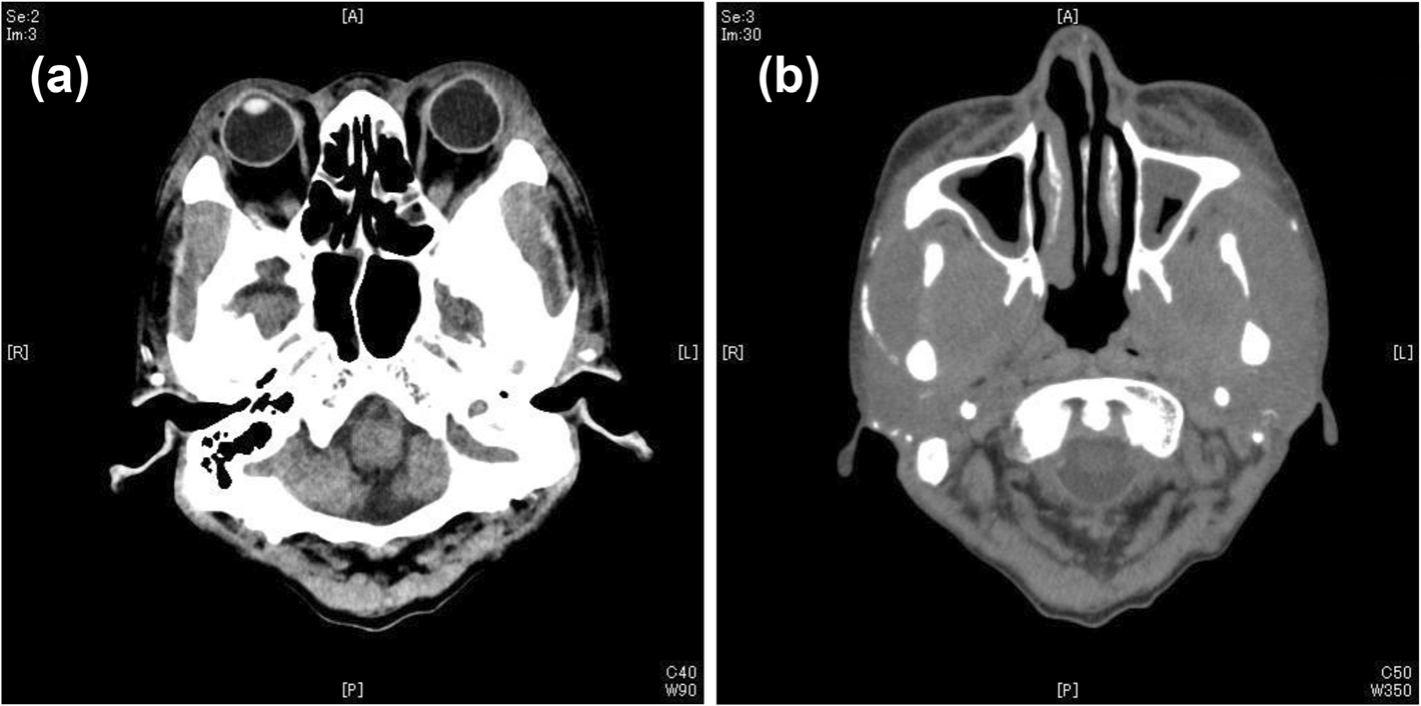
**Computed tomography scan of the facial lesion about 1 month after the admission.** Computed tomography shows findings consistent with **(a)** diffuse osteosclerotic change on orbits and skull base and peri-orbit soft tissue swelling, **(b)** mucous thickening of maxillary sinus and marked swelling of masseter.

A CT scan of the lesion showed findings consistent with osteosclerotic change on mandible, orbits and skull base, mucous thickening of maxillary sinus, and masseter muscle swelling with calcification (Figure 2).

His blood test was noted for elevated alkaline phosphatase level (1164mg/dL) and C-reactive protein (14.0mg/dL). His serum IgG4 level was within normal range (10.4mg/dL). Liposomal amphotericin B was added to voriconazole again, but his symptoms continued to worsen. He became febrile, and bilateral exophthalmos with eyelid swelling developed. Because of the deterioration of the patient’s systemic condition, empiric steroid therapy (prednisolone 40mg/day) was started to control the severe systematic inflammation. Five days after starting the steroid, biopsy was performed under general anesthesia on day 30 after the admission, and samples were taken from every site where pain or inflammation was observed (mandibular bone, maxillary sinus mucous, masseter muscle and cervical lymph nodes). In the biopsy specimens of maxillary and mandibular bones, trabeculae were lined by osteoblasts showing active bone remodeling, and intertrabecular spaces were filled by fine fibrous tissue with mild lymphocytic infiltration. Around the facial artery, there was dense fibrosis of which the histological findings were similar to those of the previous operation. Nasal mucosa was edematous and mild lymphocytic and plasmacytic infiltration was seen. Immunohistochemistry for IgG4 was performed in bone, soft tissue around the facial artery, and nasal mucosa, and positive cells were very few in every specimen (Figure 1).

After a lengthy time-course, the nature of recurring chronic inflammation at head and neck, unresponsiveness to antifungal medication and fibrotic inflammatory pathologic findings, we finally concluded that the diagnosis was TFIL. His symptoms resolved within a week and the patient was discharged home on oral steroid maintenance therapy.

## Discussion

This case report presents a case of TFIL, which remained undiagnosed for about 20 years. Exophthalmos and trismus were probably caused by the effect of inflammation of mandibular bone and orbital bones. The patient had been treated for “chronic osteomyelitis” repeatedly to no avail. We hypothesized initially that he had been suffering from chronic osteomyelitis caused by *Aspergillus*, since he was assumed to be immunocompromised by having diabetes mellitus, and a galactomannan assay was positive. Galactomannan assay is sensitive and specific in diagnosing invasive aspergillosis (79 to 96%, 74 to 99% respectively), but the role of this assay in chronic *Aspergillus* infection is not well established [8]. Galactomannan assay is also known to cause false-positive results, particularly in patients who receive penicillins or other fungal infections. A false-positive result may also occur without any known causes [8]. We continued antifungal medications since chronic *Aspergillus* infection may not respond well to the antifungal medications. However, the recurring nature of longstanding illness, failure to identify the causative organisms by repeated cultures and histological examinations, and multiple site osteomyelitis (peri-orbital and mandible) were not very consistent with this diagnosis.

Because of the chronic inflammatory nature, we also considered diagnoses such as tuberculosis; sarcoidosis; varieties of vasculitis; relapsing polychondritis; SAPHO (synovitis, acne, pustulosis, hyperostosis and osteitis) syndrome; histiocytosis X; and Erdheim–Chester disease [9], but clinical presentation and histological findings were not consistent with these disorders.

The diagnosis of TFIL is mainly based on clinical features and histological examinations. Clinical pictures of TFIL include lesions spreading to almost all areas of head and neck, sometimes (approximately 20%) accompanied by retroperitoneal or mediastinum fibrosclerotic lesion, orbital pseudotumor or Riedel’s thyroiditis. Macroscopically, these lesions are firm and tannish-white to gray-white and sometimes show a locally invasive nature like malignancy.

At histology, they appear benign and composed of mature fibrous tissue sparsely interspersed with normal-appearing fibroblasts, lymphocytes, and a few polymorphonuclear cells [10]. The lesions frequently extend into adjacent soft tissue and may encase blood vessels, nerves, and ducts.

In our case, the clinical picture and the macroscopic and microscopic features were consistent with these diagnostic criteria, and we finally made the diagnosis of TFIL.

Recent literature suggests several inflammatory diseases including TFIL may be a variation of IgG4-related disease [11]. IgG4-related disease is relatively common in Japan and thousands of patients may develop this disorder annually [12]. Elevated concentrations of IgG4 in serum and massive infiltration of IgG4+ plasma cells in the lesion are characteristic of IgG4-related disease and are helpful in diagnosing it [11,13]. Unfortunately, these markers are not specific diagnostic markers and specific pathological findings are more essential for diagnosis than negative marker findings [11]. In our case, serological markers were negative, and no specific histological findings suggesting IgG4-related disease were seen (such as dense lymphoplasmacytic infiltrate, obliterative phlebitis or eosinophil infiltrate). Therefore, we do not consider that our case was one variation of IgG4-related diseases.

Very few cases of TFIL have been reported in Japan [14], but it is possible that TFIL is more common than thought and cases may have been categorized as one variation of IgG4-related disease, or may have remained undiagnosed.

Idiopathic orbital inflammation is another disorder, which appeared similar to the current case, and it may also be associated with IgG4-related disease [15]. The presence of a mandibular lesion in our case is, however, not consistent with this disorder.

Treatment of TFIL remains controversial. A corticosteroid, such as prednisolone 0.6 to 1.0mg/kg/day tapering for several months, is effective to some extent. If the patient is suffering from compression symptoms, surgical intervention is needed. In some cases, radiation therapy is added to medical or surgical therapy [1]. Despite the benign nature of this lesion, recurrence and disease persistence are common. These patients require long-term follow-up and also may require corticosteroids maintenance therapy for months to years.

In our case, the patient did not respond well to the steroid therapies provided twice before. This may have reflected the rather short duration of the treatment, but also may have reflected the rather unpredictable nature of clinical response to the treatment in TFIL.

Better understanding of the pathophysiology of TFIL, particularly in relation to IgG4-related disease, should be elucidated by further studies.

## Conclusions

TFIL can be a considerable diagnostic challenge. Years of diagnostic work up and management were needed for reaching a conclusive diagnosis of TFIL in our case.

## Consent

Written informed consent was obtained from the patient for publication of this case report and any accompanying images. A copy of the written consent is available for review by the Editor-in-Chief of this journal.

## Abbreviations

CT: Computed tomography; Ig: Immunoglobulin; OD: Optical density; TFIL: Tumefactive fibroinflammatory lesion.

## Competing interests

The authors declare that they have no competing interests.

## Authors’ contributions

SK took care of the patient and wrote the initial draft. KI, FK, CO, YS and SN edited the manuscript with literature review. All the authors read and approved the final manuscript.
